# The complete chloroplast genomes of *Camellia chrysanthoides* and *Camellia achrysantha*

**DOI:** 10.1080/23802359.2021.1958715

**Published:** 2021-07-27

**Authors:** Yanchi Lai, Shaoqing Tang

**Affiliations:** a Key Laboratory of Ecology of Rare and Endangered Species and Environmental Protection, Ministry of Education, Guangxi Normal University, Guilin, China; b Guangxi Key Laboratory of Rare and Endangered Animal Ecology, College of Life Science, Guangxi Normal University, Guilin, China

**Keywords:** *Camellia chrysanthoides*, *Camellia achrysantha*, complete chloroplast sequence

## Abstract

*Camellia chrysanthoides* H. T. Chang and *Camellia achrysantha* H. T. Chang et S. Y. Liang are two threatened yellow camellia species endemic to southwestern Guangxi, China. Here, we report the complete chloroplast (cp) genomes of *C. chrysanthoides* and *C. achrysantha* for the first time. The total cp genome of *C. chrysanthoides* is 156,959 bp and contains a large single-copy (LSC, 86,564 bp) region, a small single-copy (SSC, 18,267 bp) region, and a pair of inverted repeat (IR, 26,064 bp) regions. The cp genome of *C. achrysantha* is 156,658 bp and includes an LSC region of 86,249 bp, SSC region of 18,243 bp, and two IR regions of 26,083 bp each. Both *C. chrysanthoides* and *C. achrysantha* have 136 genes, including 93 protein-coding genes, 35 tRNA genes, and eight rRNA genes.

*Camellia chrysanthoides* H. T. Chang *and Camellia achrysantha* H. T. Chang et S. Y. Liang (family Theaceae) have yellow flowers and are endemic to southwestern Guangxi, China. *C. chrysanthoides* grows exclusively in acidic soils and has a narrow distribution in Longzhou County (Chang 1979). It has been listed as an endangered (EN) species in the Threatened Species List of China's Higher Plants (Qin et al. 2017). *C. achrysantha* grows in calcareous soil and currently only occurs in Tuolu Town, Chongzuo, Guangxi, China. It is a valuable ornamental plant and genetic resource for breeding (Liang 1994). *C. achrysantha* is threatened because of illegal transplanting. Here, we report the complete chloroplast (cp) genomes of *C. chrysanthoides* and *C. achrysantha* to provide genetic data to support the conservation and utilization of these two rare species.

Fresh leaves of *C. chrysanthoides* and *C. achrysantha* were collected from Daqing Mountain, Longzhou County, Guangxi, China (22.30°N, 106.74°E) and Tuolu Town, Chongzuo, Guangxi, China (22.70°N, 107.72°E), respectively. Voucher specimens of *C. chrysanthoides* and *C. achrysantha* were deposited at the Herbarium of Guangxi Institute of Botany (http://ibk.gxib.cn/, Chunrui Lin, chunruilin@tom.com) under the voucher numbers IBK00430874 and IBK00430873, respectively. Genome sequencing was conducted on the Illumina NovaSeq 6000 Platform. Approximately 5.2 Gb of C. *chrysanthoides* and 5.3 Gb of *C. achrysantha* raw reads were obtained. After filtering the low-quality data, ∼5 Gb of clean data were yielded for each species. The trimmed reads were assembled by NOVOPlasty (Dierckxsens et al. 2017), and assembled genomes were annotated by PGA (Qu et al. 2019) using the complete cp genome of *Camellia impressinervis* Chang et S. Y. Liang as the reference (GenBank accession number: NC022461).

The complete cp genome sequence of *C. chrysanthoides* was 156,959 bp in length with a GC content of 37.3%; it had a typical four-conjoined structure: a large single-copy (LSC) region of 86,564 bp, a small single-copy (SSC) region of 18,267 bp, and two inverted repeats (IR) regions, each of 26,064 bp. A total of 136 genes were annotated, including 93 protein-coding genes (PCGs), eight ribosomal RNA (rRNA) genes, and 35 transfer RNA (tRNA) genes. The complete cp genome of *C. achrysantha* was similar, with a total length of 156,658 bp (37.3% GC content), including an LSC region of 86,249 bp, SSC region of 18,243 bp, and two IR regions of 26,083 bp each. It contains 136 genes, including 93 PCGs, eight rRNA genes, and 35 tRNA genes.

Phylogenomic analysis based on the complete cp genomes was performed using *C. chrysanthoides*, *C. achrysantha*, and other cp genome sequences of *Camellia. Polyspora axillaris* was used as the outgroup. The GenBank accession numbers of the sequences used in this paper are given in Figure 1. The sequence alignment was conducted using MAFFT (Katoh and Standley 2013). The phylogenetic tree was constructed using IQTREE v1.6.12 (Nguyen et al. 2015), the K3Pu + F + 1 model, and 1000 bootstrap replicates. The ML tree showed that *C. chrysanthoides* was clustered with *C. mingii* and *C. liberofilamenta* with 72% bootstrap support (Figure 1). *C. achrysantha* was closely related to *C. indochinensis* with 99% bootstrap support (Figure 1). Future studies of maternally inherited genetic information in yellow camellias are needed, and rare species of *Camellia* require protection.

**Figure 1.**
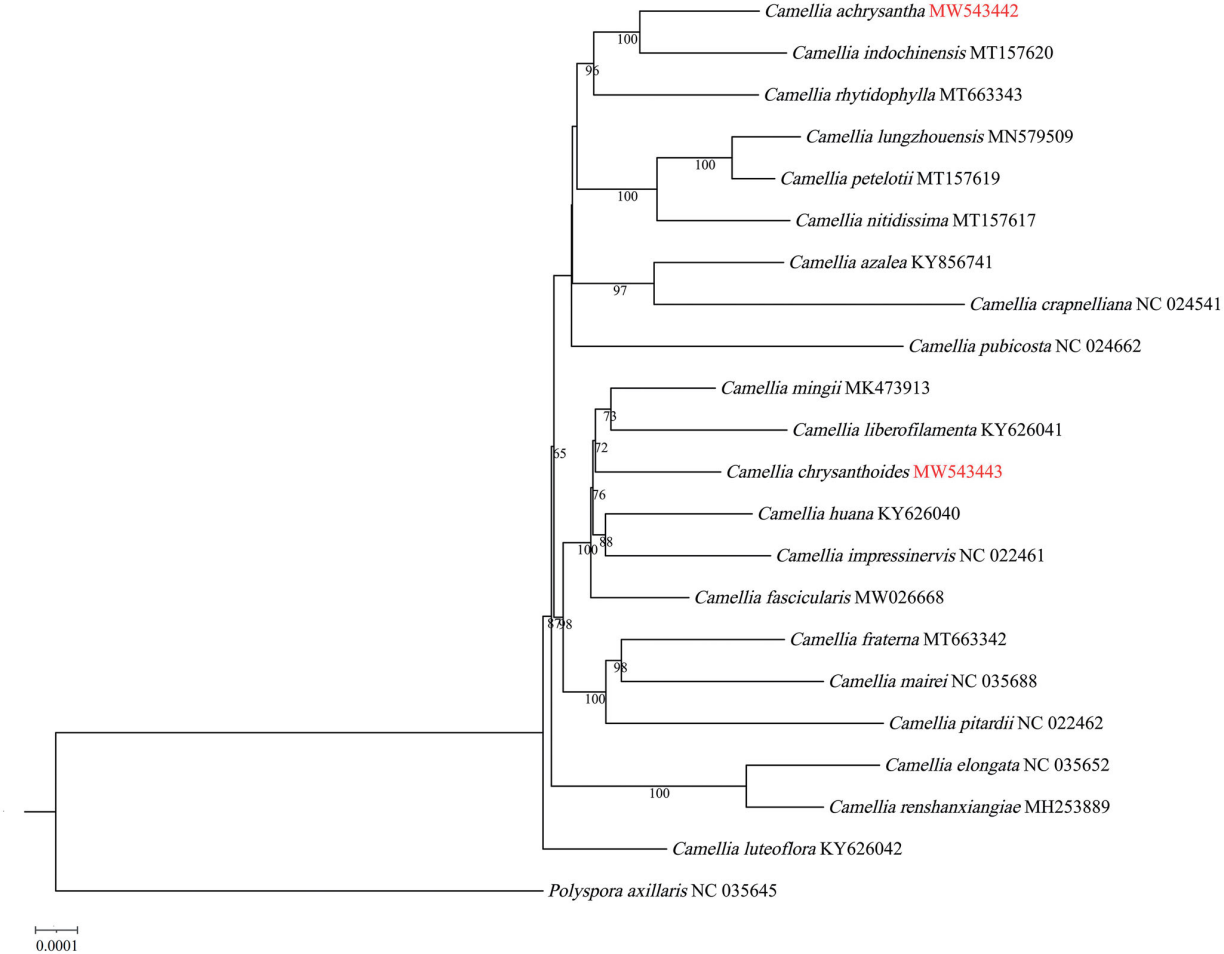
The maximum-likelihood phylogenetic tree inferred from the 22 chloroplast genome sequences of *Camellia*.

## Data Availability

The genome sequence data that support the findings of this study are openly available in GenBank of NCBI at https://www.ncbi.nlm.nih.gov/ under the accession numbers MW543442 and MW543443. The associated BioProject, SRA, and Bio-Sample numbers of *C. chrysanthoides* are PRJNA695023, SRR13636540, and SAMN17598210, respectively. The associated BioProject, SRA, and Bio-Sample numbers of *C. achrysantha* are PRJNA695023, SRR13636539, and SAMN17598211, respectively.

## References

[CIT0001] Chang HT. 1979. *Chrysantha*, a section of golden camellias from Cathaysian flora. Act Sci Nat Univ Sunyats. 3:69–74.

[CIT0002] Dierckxsens N, Mardulyn P, Smits G. 2017. NOVOPlasty: *de novo* assembly of organelle genomes from whole genome DNA. Nucleic Acids Res. 45:e18. 28204566 10.1093/nar/gkw955 PMC5389512

[CIT0003] Katoh K, Standley DM. 2013. MAFFT multiple sequence alignment software version 7: improvements in performance and usability. Mol Biol Evol. 30(4):772–780. 23329690 10.1093/molbev/mst010 PMC3603318

[CIT0004] Liang SY. 1994. *Camellia achrysantha*, a new species of yellow camellias from Fusui (Guangxi). Guangxi Forestry Sci. 23(1):52–53.

[CIT0005] Nguyen LT, Schmidt HA, von Haeseler A, Minh BQ. 2015. IQ-TREE: a fast and effective stochastic algorithm for estimating maximum-likelihood phylogenies. Mol Biol Evol. 32(1):268–274. 25371430 10.1093/molbev/msu300 PMC4271533

[CIT0006] Qin H, Yang Y, Dong S, He Q, Jia Y, Zhao L, Yu S, Liu H, Liu B, Yan Y, et al. 2017. Threatened species list of China's higher plants. Biodiv Sci. 25(7):696–744.

[CIT0007] Qu XJ, Moore MJ, Li DZ, Yi TS. 2019. PGA: a software package for rapid, accurate, and flexible batch annotation of plastomes. Plant Methods. 15(1):1–12. 31139240 10.1186/s13007-019-0435-7 PMC6528300

